# 7p21.1 Microdeletion Encompassing the ACTB Gene in a Japanese Child: Longitudinal Clinical and Neuroimaging Findings

**DOI:** 10.7759/cureus.93057

**Published:** 2025-09-23

**Authors:** Koji Yokoyama, Mitsukazu Mamada

**Affiliations:** 1 Department of Pediatrics, Japanese Red Cross Wakayama Medical Center, Wakayama, JPN

**Keywords:** 7p21.1 microdeletion, 7p21.1 microdeletion encompassing actb, developmental delay, microcephaly, white matter cysts

## Abstract

We report a Japanese male with genetically confirmed 7p21.1 microdeletion encompassing ACTB, followed longitudinally from infancy to late childhood. The patient presented at 10 months of age with microcephaly, short stature, global developmental delay, bilateral esotropia, and atypical Y-shaped deep plantar creases. Early evaluations excluded congenital infections and chromosomal abnormalities. At age four years and eight months, a brain MRI revealed multiple cystic lesions in the deep white matter near the left lateral ventricular trigone and scattered punctate hyperintensities, which remained unchanged at age 11 years and 10 months. Developmental assessments demonstrated persistent cognitive impairment (developmental quotient (DQ): 73 at age two years; 65 at age seven years). He also had growth restriction (height −2.7 SD at final follow-up), without seizures or major systemic malformations. This case represents one of the few Japanese reports of 7p21.1 microdeletion encompassing ACTB with a detailed clinical course over more than a decade and highlights an uncommon neuroradiological finding, deep white matter cysts, which may broaden the recognized phenotype. Our findings underscore the diagnostic value of exome sequencing in children with syndromic developmental delay when conventional evaluations are inconclusive and the importance of careful physical examination in early infancy to detect subtle anomalies that may provide critical diagnostic clues.

## Introduction

The ACTB gene encodes β-actin (encoding actin beta), a cytoskeletal protein that plays an essential role in maintaining cellular integrity, enabling cell motility, and regulating gene expression [1,2]. Pathogenic variants in ACTB are most frequently associated with Baraitser-Winter cerebrofrontofacial syndrome (BWCFFS), which is characterized by developmental delay, craniofacial dysmorphism, and central nervous system abnormalities [2,3]. By contrast, copy number variations involving 7p21.1 encompassing ACTB are much less common, and their phenotypic spectrum remains incompletely defined [4,5]. The 7p21.1 microdeletion encompassing ACTB is an extremely rare genetic disorder, with only a limited number of reported cases to date [5-7]. Recent studies [6,7] have delineated the clinical spectrum of ACTB-related microdeletions and refined the critical 7p22.1 region. Reported features include microcephaly, short stature, mild-to-moderate intellectual disability, epilepsy, and various structural brain abnormalities [3]. Because of its rarity, detailed longitudinal clinical descriptions are scarce, and little is known about long-term neurodevelopmental outcomes [4-6]. Here, we report a Japanese case of 7p21.1 microdeletion encompassing ACTB caused by a de novo microdeletion at 7p21.1, along with a detailed longitudinal clinical and neuroimaging follow-up from infancy to late childhood. The patient presented with microcephaly, short stature, global developmental delay, and subtle structural brain abnormalities. We describe the clinical course from early infancy to late childhood, including growth hormone stimulation testing, neuroimaging findings, and neurodevelopmental follow-up through the school years. This case underscores the diagnostic challenges encountered in early childhood, the importance of excluding more common congenital syndromes, and the utility of exome-based genetic testing in children with unexplained developmental delay and syndromic features.

## Case presentation

A male child was referred to us at 10 months of age after concerns were raised during routine health checkups conducted by a local public health center. He was born at 40 weeks of gestation with a birth weight of 2450 g, consistent with small for gestational age (SGA). Apgar scores were not available, but no resuscitation or neonatal intensive care unit (NICU) admission was required, and there were no perinatal complications. Placental pathology was not available for review. Given the stable neonatal course and absence of perinatal risk factors, intrauterine growth restriction or birth-related events are unlikely to account for the subsequent neuroimaging findings or persistent short stature. During early pregnancy (10 weeks of gestation), a hemagglutination inhibition test revealed a maternal rubella antibody titer of 1:32, indicating prior immunity; no other perinatal complications were reported.

At four months of age, microcephaly, failure to thrive, and abnormal ocular movements were noted. At six months, he exhibited persistent microcephaly, poor facial expressiveness, delayed closure of the anterior fontanelle, ocular deviation, and deep plantar creases with a Y-shaped or divergent configuration. At 10 months, he was referred to our institution.

**Figure 1:**
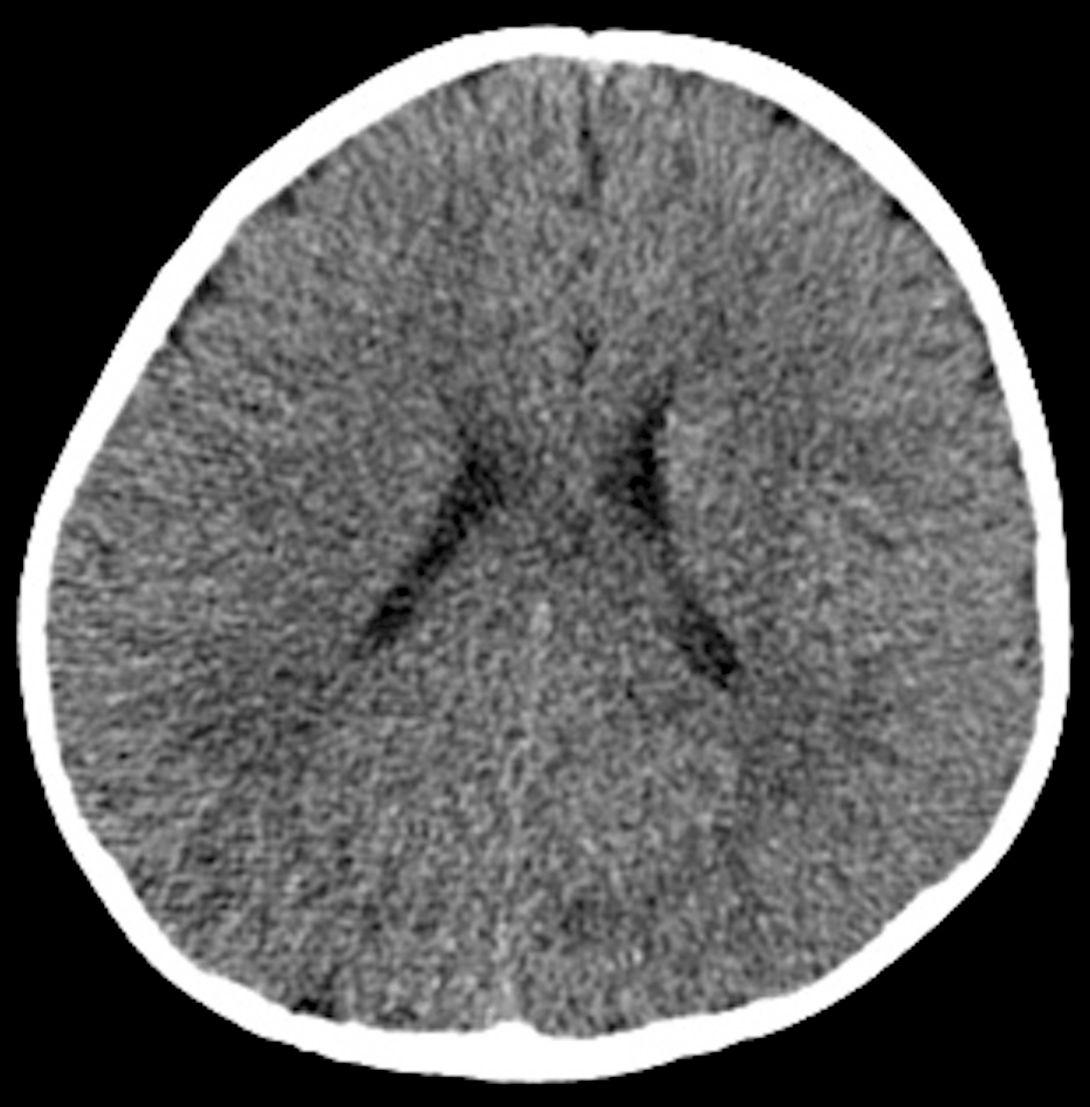
Non-contrast head CT (coronal view) at 10 months of age. No intracranial calcification, hydrocephalus, or other abnormal findings are observed.

**Figure 2:**
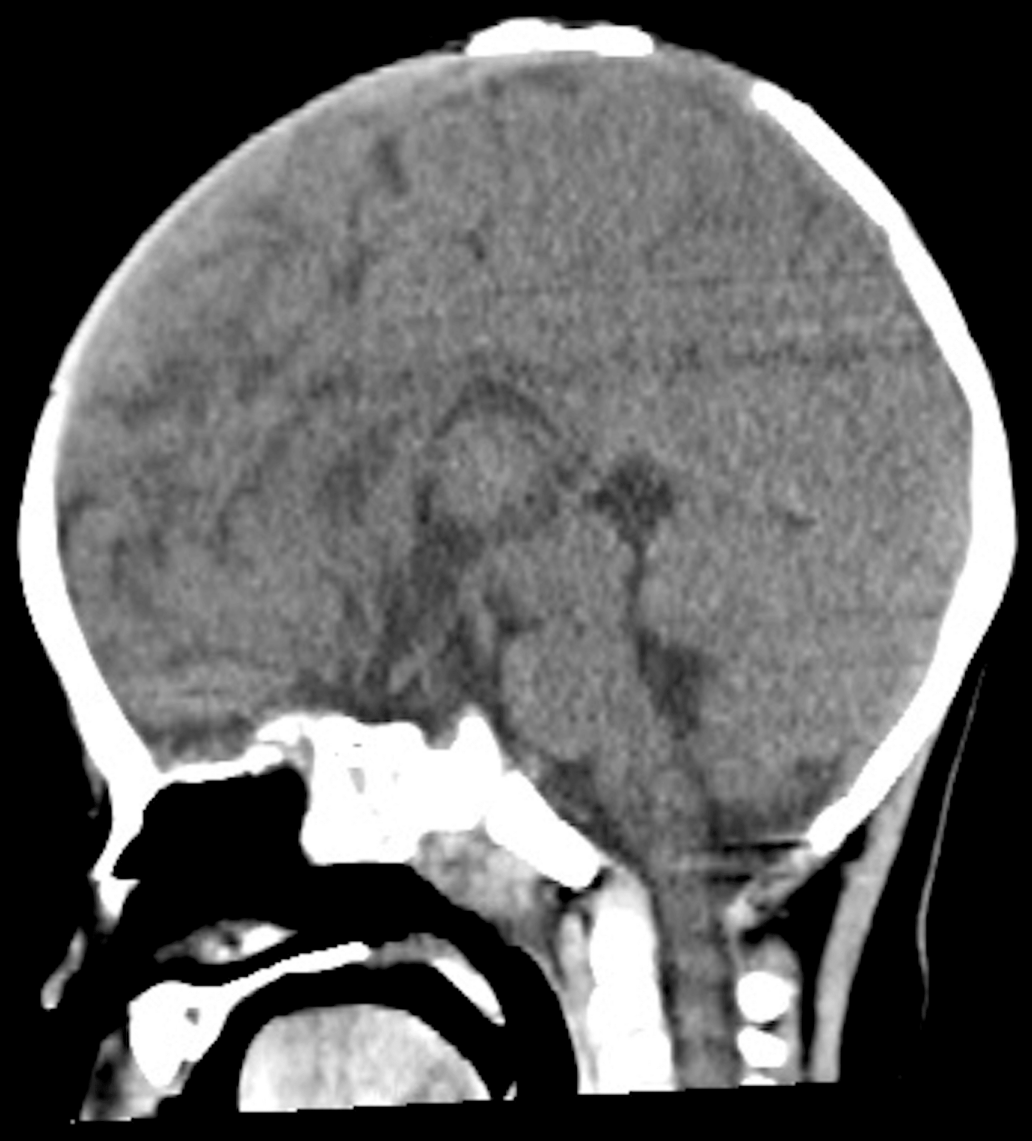
Non-contrast head CT (sagittal view) at 10 months of age. The anterior fontanelle is markedly enlarged, with delayed suture closure.

On physical examination, the anterior fontanelle measured approximately 3 × 3 cm and remained open; the posterior fontanelle was also open. Ophthalmologic evaluation was performed to rule out congenital rubella syndrome, but no salt-and-pepper retinopathy or other characteristic ocular findings were observed. The child had bilateral esotropia. His mother also had a history of esotropia and underwent corrective surgery at the age of eight. Routine cytogenetic testing of the mother revealed normal results. At that stage, the father was unavailable for testing due to estrangement. Because of the relatively high population prevalence of esotropia, the mother’s condition was considered unrelated to the patient’s suspected genetic syndrome. Abdominal and cardiac ultrasonography revealed no abnormalities. Routine blood tests and serologic tests for toxoplasmosis, rubella, cytomegalovirus, herpes, and other agents (TORCH) infections were negative. At 10 months of age, head CT showed no evidence of intracranial calcification or hydrocephalus (Figures 1, 2).

A brain MRI was not performed at that time due to clinical stability and limited availability. Cranial ultrasound was not obtained. Because a later MRI revealed multiple cystic white matter lesions, the absence of an early MRI or ultrasound makes it uncertain whether these changes were congenital or developed subsequently. Chromosomal analysis using G-banding revealed a normal karyotype. He had no history of unprovoked seizures or febrile seizures, and there were no clinical episodes suggestive of epilepsy. Although the patient exhibited early vocalizations, he had difficulty with communication and social interactions. At one year and seven months, his height was 74.0 cm (-2.5 SD), weight was 8.32 kg (-1.9 SD), and head circumference was 44.0 cm (-3.0 SD) and 45.0 cm (-2.8 SD) at two years and two months, both persistently below -2 SD, indicating microcephaly without catch-up growth. Birth and early infancy measurements were not systematically recorded. These values are summarized in Table 1.

**Table 1:** Longitudinal head circumference measurements. Available data indicate persistent microcephaly without catch-up growth, with measurements of 44.0 cm (–3.0 SD) at one year and seven months and 45.0 cm (–2.8 SD) at two years and two months. Birth and early infancy measurements were not systematically recorded.

Age	Head circumference (cm)	SD
1 y 7 m	44	-3
2 y 2 m	45	-2.8

At two years and two months, developmental assessment using the Kyoto Scale of Psychological Development 2001 (KSPD 2001) [8] revealed global developmental delay in the borderline range (overall developmental quotient (DQ) 73). At age seven years, a follow-up developmental assessment conducted by the local public health center reported a global DQ of 65. Detailed subtest scores and representative clinical observations at age two years are provided in Table 2.

**Table 2:** Results of the Kyoto Scale of Psychological Development 2001 (KSPD 2001) at age two years and two months. The table summarizes the developmental ages, developmental quotients (DQ), and representative clinical observations in each domain (Postural–Motor, Cognitive–Adaptive, and Language–Social). The assessment was conducted by a trained clinical psychologist in a standardized outpatient setting. The findings indicate borderline global developmental delay, with relatively preserved motor and adaptive skills compared to more delayed language and social development. At age seven years, a follow-up developmental assessment conducted by the local public health center reported a global DQ of 65, although detailed subtest data were not available. According to correspondence from the Kyoto International Social Welfare Exchange Center (KISWEC), no additional copyright permission was required to report only the developmental quotient (DQ) results from the Kyoto Scale of Psychological Development (KSPD).

Domain	Developmental age	DQ	Clinical observations
Postural–Motor	1 y 8 m	77	Climbed stairs with handrail; unable to jump with both feet; strong arm strength; could hang.
Cognitive–Adaptive	1 y 8 m	77	Stacked three blocks; discriminated three shapes; scribbled with weak pencil pressure.
Language–Social	1 y 5 m	73	Limited expressive words (“bu-bu” during test); at home used “mamma,” “umai”; followed simple commands; responded socially.

**Figure 3:**
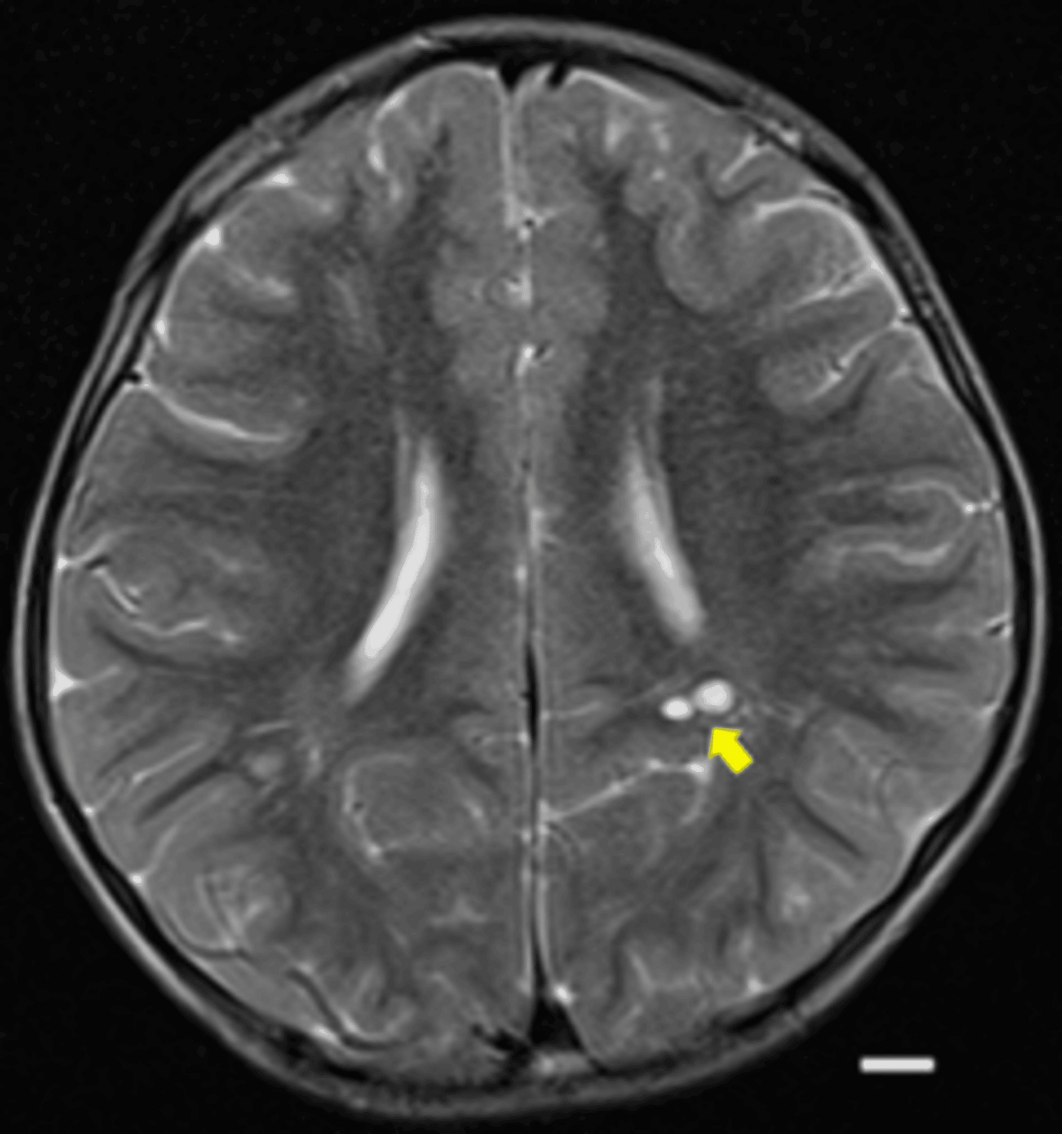
Brain MRI at four years and eight months of age, performed on a 1.5 Tesla scanner. Axial T2-weighted images (slice thickness 5 mm) demonstrate multiple cystic lesions in the deep white matter near, but not directly adjacent to, the left lateral ventricular trigone (arrow). No hemosiderin deposition was observed around the cysts. In addition, scattered punctate hyperintensities are noted in the bilateral deep white matter. Diffusion-weighted imaging (DWI) and apparent diffusion coefficient (ADC) maps showed no restricted diffusion, and susceptibility-weighted imaging (SWI) revealed no hemorrhagic or calcific lesions. Thus, no additional information beyond the T2-weighted images was obtained. Scale bar = 1 cm.

At four years and eight months, he caught up in verbal communication and was attending a regular preschool. His height was 91.7 cm (-3.1 SD), weight was 12.1 kg (-2.2 SD), and BMI was 14.3. Bone age (radius-ulna-short bones (RUS) method) was delayed by 3.2 years or younger. Serum IGF-1 was 42 ng/mL (-1.6 SD). At four years and eight months, growth hormone (GH) stimulation testing was performed using both growth hormone-releasing peptide-2 (GHRP2) and intravenous arginine, in accordance with the Japanese standard practice of dual stimulation testing. For the GHRP2 test, 2 µg/kg was administered with blood samples collected over 60 minutes; the diagnostic cutoff for GH deficiency was defined as a peak <16 ng/mL. For the arginine test, 0.5 g/kg was administered with blood samples collected over 120 minutes; the diagnostic cutoff was defined as a peak <6 ng/mL. Serum GH was measured using a chemiluminescent immunoassay (CLIA). The patient achieved a peak GH level of 27.9 ng/mL, thereby clearly excluding GH deficiency. Brain magnetic resonance imaging (MRI) identified multiple cystic lesions in the deep white matter near the left lateral ventricle trigone (Figure 3).

At five years and three months, whole-exome sequencing (WES) was performed using the Illumina HiSeq 2500 platform (Illumina, Inc., CA, USA) with an average on-target coverage of approximately 100×. Copy-number analysis was conducted with an in-house pipeline based on read-depth profiling, which identified a presumed de novo heterozygous microdeletion spanning chr7:6,243,891-7,889,083 (GRCh37/hg19). The predicted deletion was subsequently confirmed by quantitative polymerase chain reaction (qPCR), thereby validating the result. This 1.65 Mb loss encompassed ACTB as well as additional genes (POLR2J2, RASA4, RNF216). The variant has not yet been deposited in ClinVar, but deposition is in progress. The mother’s chromosomal testing was normal; however, paternal DNA was unavailable, so the de novo status could not be fully confirmed. Based on prior reports of patients with an identical deletion [7], the deleted interval spans chr7:6,243,891-7,889,083 (GRCh37/hg19), approximately 1.65 Mb in size, and includes several genes such as ACTB, POLR2J2, RASA4, and RNF216. This confirmed the diagnosis of 7p21.1 microdeletion encompassing ACTB.

**Figure 4:**
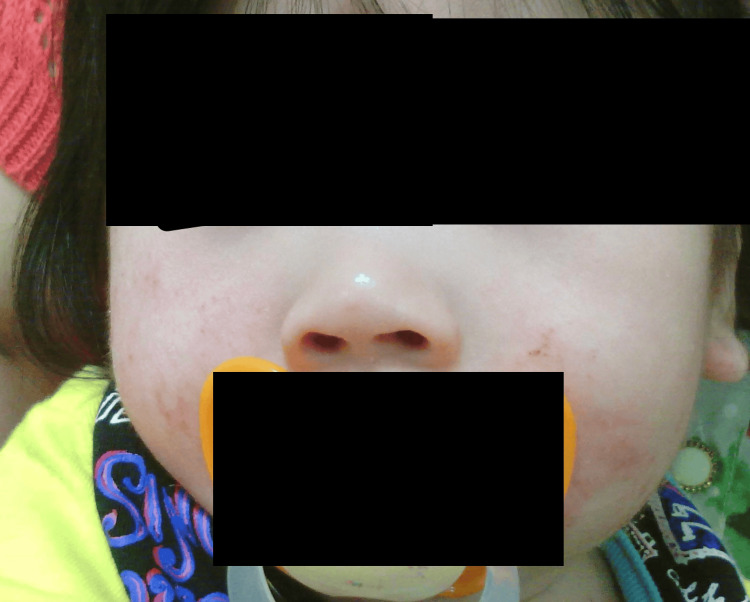
Facial appearance at nine months of age. Mild dysmorphic features are present, including a broad nasal bridge and flat midface. The eye region and eyebrows have been obscured to protect patient privacy.

**Figure 5:**
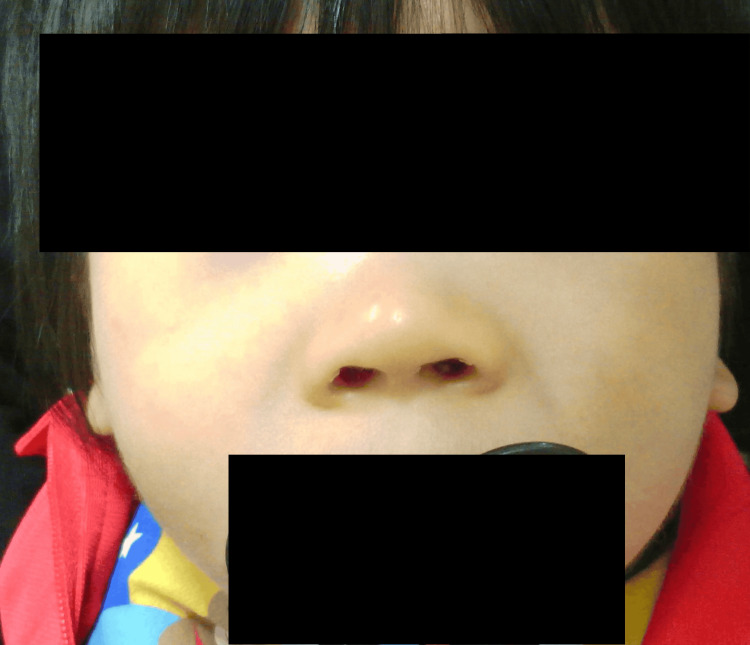
Facial appearance at one year and six months of age. Mild dysmorphic features persist, with gradual evolution of facial morphology. The eye region and eyebrows have been obscured to protect patient privacy.

**Figure 6:**
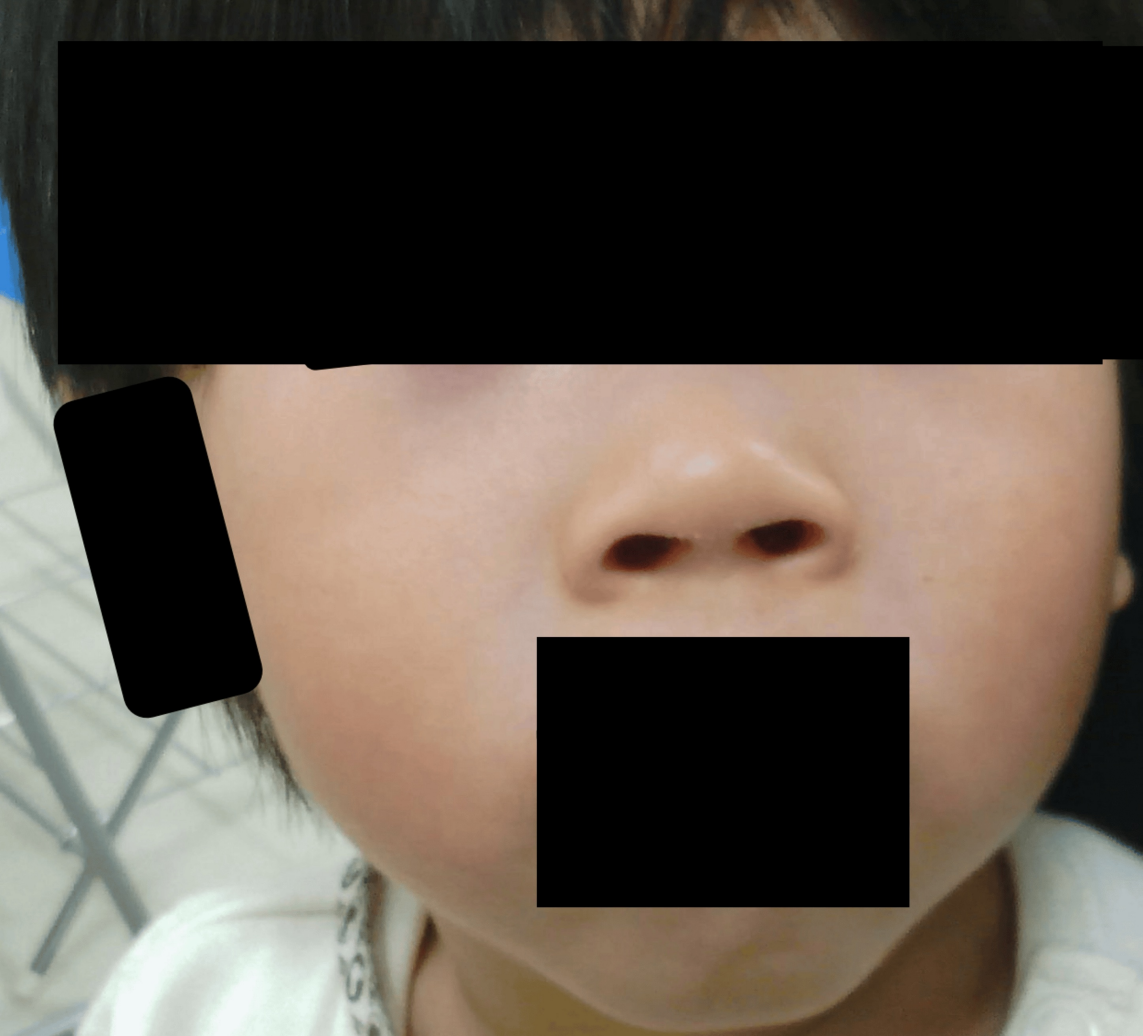
Facial appearance at two years and eight months of age. Dysmorphic features remain mild, with subtle changes compared to earlier photographs. The eye region, eyebrows, ears, and lips have been obscured to protect patient privacy.

Facial photographs were obtained at age nine months (Figure 4), one year and six months (Figure 5), and two years and eight months (Figure 6). Notable features included a broad nasal bridge and strabismus, whereas epicanthal folds, low-set ears, and a thin upper lip were absent. These findings were mild and evolved gradually over time. In the figure, the eyes have been obscured to protect privacy. The patient's family provided written informed consent to participate and publish. To the best of our knowledge, this is one of the few Japanese cases of 7p21.1 microdeletion encompassing ACTB reported to date, and it is among the rare examples with a documented clinical course spanning more than a decade.

**Figure 7:**
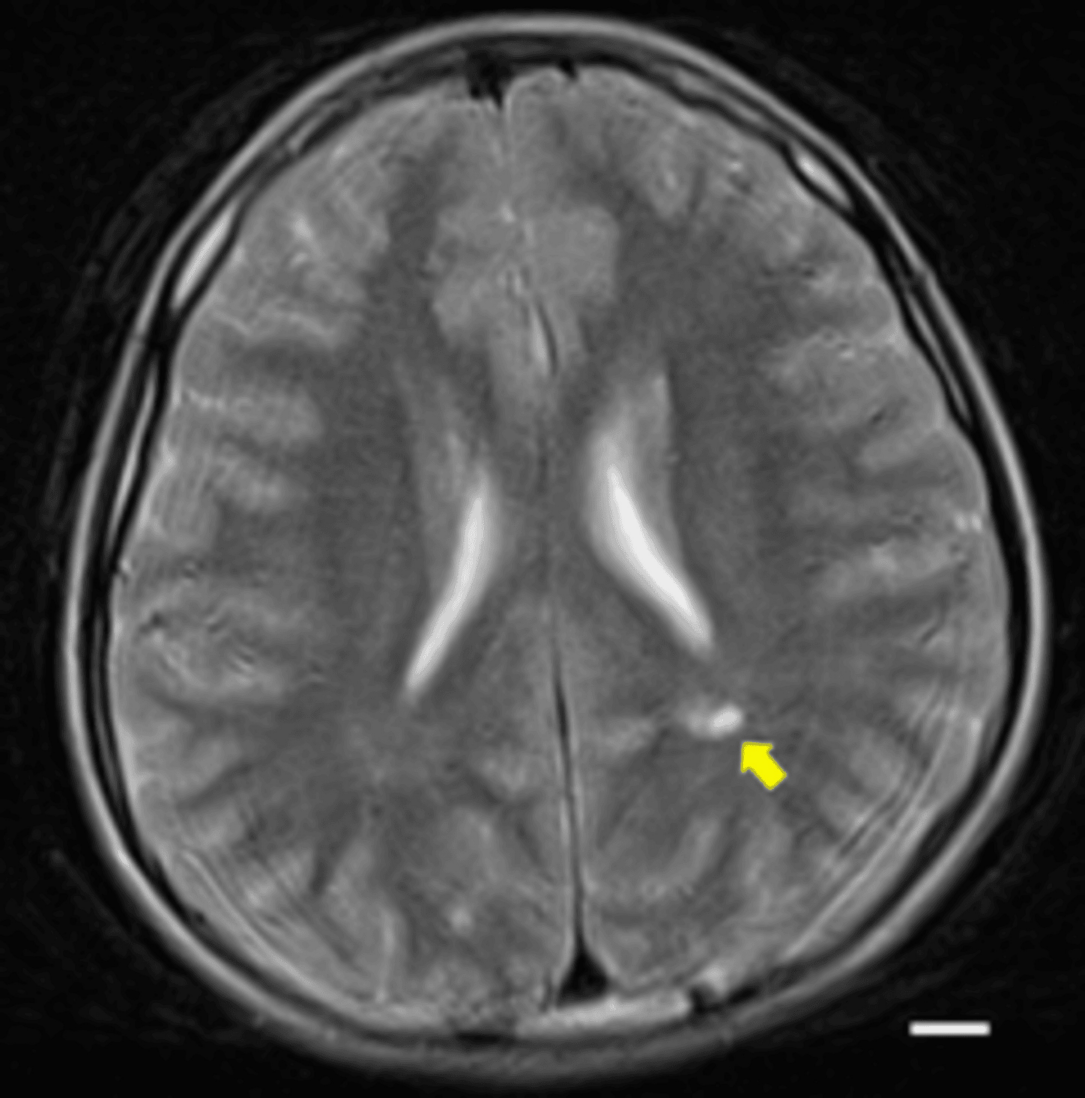
Brain MRI at 11 years and 10 months of age, performed on a 1.5 Tesla scanner. Axial T2-weighted images (slice thickness 5 mm) show a cystic lesion in the deep white matter of the left parietal region near the lateral ventricular trigone (arrow). The lesion measured approximately 10 mm in maximal diameter at both four years and eight months and 11 years 10 months, indicating no significant interval change. No progression of punctate hyperintense areas is observed. Diffusion-weighted imaging (DWI) and apparent diffusion coefficient (ADC) maps showed no restricted diffusion, and susceptibility-weighted imaging (SWI) revealed no hemorrhagic or calcific lesions. A white scale bar indicates 10 mm.

At age seven, the patient's DQ was 65, and he began attending a special needs class in a public elementary school. At his most recent follow-up at 11 years and 10 months, he continued to attend special education classes. His height was 127.8 cm (-2.7 SD), weight was 24 kg (-1.8 SD), and BMI was 14.7. He was receiving psychosocial support for impulsive behavior. The brain MRI findings first noted at age four (i.e., cystic lesions and punctate white matter hyperintensities) remained unchanged at 11 years and 10 months (Figure 7).

This case report was reviewed and approved by the Ethics Committee of the Japanese Red Cross Wakayama Medical Center (approval number: 1527; approval date: August 22, 2025). Written informed consent for publication was obtained from the patient’s legal guardians, including consent for the use of facial photographs with anonymization and for the sharing of genetic findings. Consent for deposition of the genetic variant into public databases (ClinVar) was also obtained. 

## Discussion

7p21.1 microdeletion encompassing ACTB is an extremely rare genetic disorder caused by haploinsufficiency of the ACTB gene encoding β-actin, a key cytoskeletal protein involved in cell motility, proliferation, and regulation of gene expression [1,6]. While missense and nonsense mutations in ACTB are more commonly associated with BWCFFS, which is a distinct clinical entity, microdeletions encompassing ACTB have been reported far less often, and their phenotypic spectrum remains incompletely defined [6,9]. Clinically, BWCFFS is characterized by prominent craniofacial dysmorphism (including ptosis, hypertelorism, and a broad nasal bridge), cortical malformations such as pachygyria or lissencephaly, and a high incidence of epilepsy [1,4]. By contrast, 7p21.1 microdeletion encompassing ACTB often presents with milder and less specific dysmorphic features, variable developmental delay, and white matter abnormalities without severe cortical malformations [5,6]. Reported manifestations include microcephaly, short stature, developmental delay, mild-to-moderate intellectual disability, seizures, and variable structural brain abnormalities [5,7,10]. Standardized anthropometric measurements documented microcephaly in early childhood (head circumference 44 cm at one year and seven months and 45 cm at two years and two months, both <-2 SD) and persistent short stature at 11 y 10 m (height 127.8 cm, -2.7 SD; weight 24 kg, -1.8 SD). Dysmorphic features are summarized in Table 2 and compared with the ACTB/7p microdeletion case series [6,7]. Our patient showed overlapping features such as microcephaly, short stature, and a broad nasal bridge, while lacking epicanthal folds, low-set ears, and a thin upper lip. He also presented with periventricular white matter cysts, a feature not systematically reported in previous series, but lacked the hallmark craniofacial features, cortical malformations, and seizure history typical of BWCFFS. Notably, he had no history of unprovoked or febrile seizures, and there were no clinical episodes suggestive of epilepsy, which may indicate a milder neurological phenotype within the 7p21.1 microdeletion encompassing the ACTB spectrum.

A notable strength of this report is the longitudinal follow-up from infancy to late childhood, including serial developmental assessments, neuroimaging, growth hormone evaluation, and anthropometric measurements. Initially, congenital infections such as congenital rubella syndrome were suspected, but these were excluded systematically through ophthalmologic, serologic, and neuroimaging evaluations. Other differential diagnoses, including chromosomal anomalies and known congenital syndromes, were ruled out via standard cytogenetic testing and clinical assessments. The brain MRI findings (multiple small cystic lesions in the deep white matter) are particularly noteworthy, as they have rarely been described in 7p21.1 microdeletion encompassing ACTB. While nonspecific white matter abnormalities have been noted in some prior reports, cystic lesions of this nature appear uncommon [5,6]. These abnormalities may reflect disruption of cytoskeletal dynamics during neurodevelopment, which is biologically plausible given the central role played by β-actin in cell structure and migration [7,11,12]. β-actin is a ubiquitously expressed cytoskeletal protein that not only maintains cellular morphology but also orchestrates intracellular transport, endocytosis, and cell-cell adhesion [13]. In neurons, β-actin is essential for growth cone motility, dendritic spine formation, and synaptic plasticity; thus, its haploinsufficiency can impair neuronal migration, cortical layer organization, and axonal pathfinding [12,14]. Such defects may plausibly contribute to altered white matter development, although this remains speculative in the absence of direct evidence from neuropathological or animal studies. Functional studies of ACTB haploinsufficiency have demonstrated impaired cytoskeletal organization and neuronal migration [6,7], which could provide a potential mechanistic basis for the observed white matter abnormalities in our patient. Beyond the nervous system, β-actin contributes to chondrocyte proliferation and differentiation within the growth plate [15]. Disruption of cytoskeletal integrity in these cells may impair endochondral ossification, providing a plausible explanation for the patient’s persistent short stature despite normal secretion of growth hormone [16,17]. Taken together, ACTB haploinsufficiency could represent a single upstream defect that simultaneously perturbs neural network formation and somatic growth, thereby unifying the neurodevelopmental delay, microcephaly, and short stature into a coherent pathophysiological framework [7,11]. This case also highlights the diagnostic value of exome sequencing in children with syndromic features and nonspecific early developmental delay. Conventional genetic and infectious workups failed to establish a diagnosis, whereas exome analysis identified a pathogenic microdeletion at 7p21.1 involving ACTB [18].

Based on the ACMG/ClinGen copy-number variant (CNV) standards [19], the 1.65 Mb deletion at 7p21.1 meets multiple pathogenic criteria (1A: size >1 Mb with several protein-coding genes; 2H: 7p22.1 region curated as haploinsufficient in the ClinGen Dosage Sensitivity Map; 2F: overlap with previously reported pathogenic 7p22.1 microdeletions; 2O: presumed de novo with negative maternal testing. Paternal DNA was not available, and thus we cannot fully exclude the possibility of paternal transmission or low-level parental mosaicism. Accordingly, we classify this CNV as pathogenic. Phenotypic features observed in our patient (e.g., broad nasal bridge, strabismus) are variably reported in the ACTB/7p deletion series and are summarized comparatively in Table 3; we therefore do not regard any single minor anomaly as a diagnostic discriminator [20,21].

**Table 3:** Dysmorphic features in our patient compared with previously reported ACTB/7p microdeletion cases. The table summarizes standardized anthropometric findings and craniofacial features in our patient, alongside their frequency in previously published case series [6,7]. Our patient showed microcephaly, short stature, developmental delay, strabismus, and a broad nasal bridge, while lacking epicanthal folds, low-set ears, and a thin upper lip. White matter cysts were also observed, a finding not systematically reported in earlier series.

Feature	Present in patient	Reported in Cuvertino 2017 [7]	Reported in Palumbo 2018 [6]
Microcephaly	Yes (HC 44 cm at 1 y 7 m; 45 cm at 2 y 2 m, both < –2 SD)	Common	Common
Short stature	Yes (Ht 127.8 cm at 11 y 10 m, –2.7 SD; Wt 24 kg, –1.8 SD)	Common	Common
Global developmental delay	Yes	Common	Common
Strabismus	Yes	Reported in the subset	Reported in the subset
Broad nasal bridge	Yes	Frequent	Frequent
Epicanthal folds	No	Frequent	Variable
Low-set/posteriorly rotated ears	No	Frequent	Frequent
Thin/arched upper lip	No	Variable	Reported
White matter cysts	Yes	Not systematically reported	Not systematically reported

Finally, we observed a Y-shaped plantar crease. This feature has not been systematically reported in the ACTB/7p deletion case series, and similar minor dermatoglyphic anomalies can occasionally be seen in other syndromes or in the general population. Therefore, we present it here descriptively but do not consider it to have diagnostic specificity [22].

To the best of our knowledge, this is one of the few Japanese cases of 7p21.1 microdeletion encompassing ACTB reported to date, and it is among the rare examples with a detailed longitudinal clinical course from infancy to adolescence. Because phenotypic expression may vary by ancestry, the inclusion of serial facial photographs in this report may aid future multi-ethnic comparative studies and help to broaden our understanding of the phenotypic spectrum of ACTB deletion syndrome.

## Conclusions

In conclusion, we report a Japanese child with a 7p21.1 microdeletion encompassing ACTB, highlighting longitudinal neurodevelopmental and neuroimaging findings that broaden the clinical spectrum of this rare disorder. This report is strengthened by providing definitive genomic coordinates with orthogonal qPCR validation, a clear statement regarding presumed de novo status, detailed MRI technical parameters and quantitative lesion measurements, more granular developmental assessment results, and a formal ACMG/ClinGen CNV classification with planned ClinVar deposition.
